# Exosomal miR‐130a‐3p regulates osteogenic differentiation of Human Adipose‐Derived stem cells through mediating SIRT7/Wnt/β‐catenin axis

**DOI:** 10.1111/cpr.12890

**Published:** 2020-08-17

**Authors:** Shude Yang, Shu Guo, Shuang Tong, Xu Sun

**Affiliations:** ^1^ Department of Plastic Surgery The First Hospital of China Medical University Shenyang China

**Keywords:** Adipose‐derived stem cells, bone regeneration, exosomes, miR‐130a‐3p, Wnt/β‐catenin

## Abstract

**Objectives:**

It is of profound significance for clinical bone regeneration to clarify the specific molecular mechanism from which we found that osteogenic differentiation of adipose‐derived stem cells (ADSCs) will be probably promoted by exosomes.

**Materials and Methods:**

By means of lentiviral transfection, miR‐130a‐3p overexpression and knockdown ADSCs were constructed. Alizarin Red S was used to detect the calcium deposits, and qPCR was used to detect osteogenesis‐related genes, to verify the effect of miR‐130a‐3p on the osteogenic differentiation of ADSCs. CCK‐8 was used to detect the effect of miR‐130a‐3p on the proliferation of ADSCs. The target binding between miR‐130a‐3p and SIRT7 was verified by dual‐luciferase reporter gene assay. Furthermore, the role of Wnt signalling pathway in the regulation of ADSCs osteogenesis and differentiation by miR‐130a‐3p was further verified by detecting osteogenic‐related genes and proteins and alkaline phosphatase activity.

**Results:**

(a) Overexpression of miR‐130a‐3p can enhance the osteogenic differentiation of ADSCs while reducing protein and mRNA levels of SIRT7, a target of miR‐130a‐3p. (b) Our study further found that overexpression of miR‐130a‐3p leads to down‐regulation of SIRT7 expression with up‐regulation of Wnt signalling pathway‐associated protein. (c) Overexpression of miR‐130a‐3p inhibited proliferation of ADSCs, while knockdown promoted it.

**Conclusions:**

The obtained findings indicate that exosomal miR‐130a‐3p can promote osteogenic differentiation of ADSCs partly by mediating SIRT7/Wnt/β‐catenin axis, which will hence promote the application of exosomal microRNA in the field of bone regeneration.

## Graphical abstract

In order to promote the application of exosomal microRNA in the field of bone regeneration, we investigate herein whether exosomal miR‐130a‐3p can promote osteogenic differentiation of ADSCs partly by mediating SIRT7/Wnt/β‐catenin axis. When exosomes fuse with the cell membrane, the 'cargos' are released. Enriched miR‐130a‐3p in exosomes binds to SIRT7 mRNA, resulting in SIRT7 mRNA degradation. Eventually, Wnt signaling pathway changes from 'off state' to 'on state', initiating the osteogenic differentiation process of ADSCs.

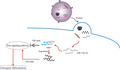

## 1 INTRODUCTION

Regeneration and repair of bone tissue are pluripotent stem cells through a series of complex process completed. It mainly includes proliferation and differentiation, recognition of extracellular matrix and signal molecules, expression of related factors and targeting. Adipose‐derived stem cells (ADSCs) are a population of non‐hematopoietic adult stem cells with self‐renewal ability and multi‐lineage potential isolated from adipose tissue.^1^, ^2^ Compared with other adult stem cells, ADSCs can not only differentiate into adipocytes, chondrocytes and osteoblast, but also have the advantages of abundant storage in vivo, easy acquisition and expansion.^3^ In recent years, the application of ADSCs in the field of bone regeneration has received extensive attention.

Achieving directional osteogenic differentiation of stem cells is the key to bone regeneration, and 'inducing factors' play a critical role in this process. Our previous studies have confirmed that the exosomes derived from osteogenically differentiated ADSCs can be used as an effective 'inducing factor' to promote osteogenic differentiation of ADSCs.^4^ However, the molecular mechanism of exosomes in the osteogenic differentiation of ADSCs remains elusive. MicroRNAs (miRNAs) are endogenous non‐protein‐coding RNA with a length of about 22 nt, which is a quintessential post‐transcriptional regulator and it can regulate the expression of target genes mainly through specific binding with 3'UTR of target genes.^5^ As an important 'cargo' of exosomes, miRNAs have been demonstrated to regulate bone regeneration.^6^, ^7^, ^8^ In our previous studies, we intended to figure out how miRNAs regulate bone regeneration, so we used microarray assays to analyse the different expression of exosomal miRNAs, and found that miR‐130a‐3p has the highest fold change, which means that miR‐130a‐3p may be the main influence.^4^ So how does miR‐130a‐3p play a part as a regulator? Xiao F et al found that miR‐130a‐3p can significantly alleviate fatty liver, which mainly regulates liver lipid metabolism by regulating FAS expression.^9^ In addition, it was also noted that the treatment of 3T3‐L1 cells with miR‐130a‐3p could reduce lipid production.^10^ Up to this date, other researches on miR‐130a‐3p are more focused on the field of cancer.^11^, ^12^ However, the role of miR‐130a‐3p in osteogenic differentiation of mesenchymal stem cells (MSCs) is rarely known.

As a member of the sirtuin family of NAD^+^‐dependent deacetylase,^13^ SIRT7 interacts with various substrate proteins through its deacetylation activity and participates in the regulation of cell proliferation, senescence, apoptosis, metabolism and other processes.^14^, ^15^ Currently, an increasing number of studies have indicated that sirtuin family is closely related to osteogenic differentiation.^16^, ^17^, ^18^ According to previous studies, SIRT7 has been shown to promote adipogenesis,^19^ while inhibiting osteogenic differentiation of bone marrow mesenchymal stem cells (BMSCs).^20^ Moreover, as mentioned before, miRNAs are considered to be an endogenous regulator of SIRT7, which binds to 3'UTR of SIRT7 to down‐regulate its expression.^21^

In the past few years, many members of the sirtuin family have been confirmed to relate to Wnt signalling pathway closely.^20^, ^22^, ^23^ Wnt signalling pathway has been widely confirmed to play an important role in the osteogenic differentiation of MSCs. Among them, the canonical Wnt signalling pathway is characterized by stable expression of β‐catenin and its transfer into the nucleus. When in the absence of Wnt ligands, β‐catenin interacts with Axin, adenomatous polyposis coli (APC), casein kinase 1 (CK1) and glycogen synthase kinase 3β (GSK‐3β) to form 'APC‐Axin‐GSK‐3β' complex. As a result, β‐catenin is degraded and cannot enter the nucleus, forming an 'off‐state' pathway. When the expression of Wnt ligands is activated, Wnt interacts with lipoprotein receptor‐related protein (LRP) and Frizzled to form receptor complex, and loose protein (Dsh) in the cytoplasm gathers under the cell membrane, leading to GSK‐3β phosphorylation and preventing 'APC‐Axin‐GSK‐3β' complex from formation. Thus, the degradation process of β‐catenin is blocked, and a large amount of free β‐catenin enters the nucleus, which is to say, Wnt signalling pathway is now 'on‐state'.^24^

In this study, we confirmed the vital role of exosomal miR‐130a‐3p in the osteogenic differentiation of ADSCs, as well as clarified the targeting relationship between miR‐130a‐3p and SIRT7. Furthermore, we explored the modulation of Wnt signalling pathway in this process, so as to confirm the important significance of miR‐130a‐3p/SIRT7/Wnt/β‐catenin axis in regulating the osteogenic differentiation process of ADSCs, providing a theoretical basis for bone regeneration.

## 2 MATERIALS AND METHODS

### 2.1 Isolation, culture and characterization of ADSCs

According to methods previously reported,^25^, ^26^ ADSCs were isolated from human adipose tissue obtained from patients who were undergoing liposuction at the Department of Plastic Surgery, the First Hospital of China Medical University. Adherent cells were cultured in a growth medium [DME F12 (HyClone, USA), 10% FBS, 1% Penicillin‐Streptomycin Solution (Gibco, USA)] at 37°C/5% CO2 and saturated humidity. ADSCs were passaged after reaching 90% confluence.

Multi‐lineage potential assay and flow cytometry analysis were performed to identify characteristics of ADSCs, as we have done in the past.^4^ The antibodies including anti‐CD34‐FITC, anti‐CD45‐PE, anti‐CD44‐FITC, anti‐CD73‐PE and anti‐CD105‐PE were purchased from BD biosciences (USA).

### 2.2 Extraction and Identification of ADSCs‐derived exosomes

ADSCs‐derived exosomes used in this study were obtained by Differential Ultracentrifugation, as described previously by Théry C et al^27^ In order to define the exosomes, transmission electron microscopy [TEM, (HITACHI, JAPAN)] was used to observe their structures. The characteristic markers including TSG101, calnexin and CD9 were analysed by Western blot, as described in 2.8.

### 2.3 Nanoparticle tracking analysis (NTA)

The exosomes sample pool was washed with deionized water. ZetaView Particle Metrix (Particle Metrix, Germany) is calibrated with polystyrene microspheres with a size of 110 nm. Then, the sample pool was washed with 1 × PBS. Finally, the exosomes sample was diluted by 1 × PBS buffer (Biological Industries, Israel) to for testing.

### 2.4 Lentiviral transfection

The lentivirus particles used in this study were purchased from GenePharma company (China). First, 1 × 10^5^ ADSCs were plated in a 6‐well plate and incubated overnight in conventional growth medium under the condition of 37°C/5% CO2 and saturated humidity. Next, cells were incubated with lentiviral particles and 5 μg/mL polybrene in conventional growth medium at a multiplicity of infection (MOI) of 50. After 24 hours, the results were observed under a light microscope (OLYMPUS, Japan) and an inverted fluorescence microscope (OLYMPUS, Japan). Two days later, the expression of miR‐130a‐3p was measured by qPCR to see whether transfection is successful or not. It was illustrated more in Materials and Methods 2.7.

### 2.5 Cell viability assay

ADSCs were seeded in a 96‐well plate with 5000 cells/100μL in each well. According to the manufacturer's instructions, after 24, 48 and 72 hours incubation, 10 μL Cell Counting Kit‐8 [CCK8 (Beyotime, China)] was added into each well. After three more hours of incubation, the result of cell proliferation was detected by a Microplate Reader (MD SpectraMax Plus384) at a rate of 450 nm.

### 2.6 Osteogenic induction of ADSCs and Alizarin Red S (ARS) assay

When the cells reached to 90% confluence, the conventional growth medium was replaced with osteogenic differentiation medium, which was prepared by 15 mL 15% foetal bovine serum [FBS (Gibco, USA)], 10 μL 10^−8^ mol/L dexamethasone sodium phosphate (Beyotime, China), 1 mL 1.8 mmol/L KH_2_PO_4_ (Beyotime, China), 1 mL 100 U/mL penicillin (Gibco), 1 mL 100 U/mL streptomycin (Gibco), 1 mL 0.1 mmol/L L‐ascorbic acid phosphate (Beyotime, China), 1 mL 2 mmol/L Glutamine (Beyotime, China) and Alpha MEM (Hyclone, USA) to up 100 mL. The medium was renewed every 3 days till Day 7 or Day 14. Mineral deposit was then detected by ARS Staining (Cyagen, USA). According to the manufacturers’ instructions, the cells were fixed with 2 mL of 4% neutral formaldehyde solution for 30 minutes. After removing formaldehyde and washing the plate with PBS, ARS solution was added for 5 minutes. The staining cells were observed by a light microscope (OLYMPUS, Japan).

### 2.7 Alkaline phosphatase (ALP) activity assay

The detected cells were lysed by Cell lysis buffer for Western and IP without inhibitors (Beyotime Biotechnology, China), and the supernatant was collected for semi‐quantitative analysis of ALP using an Alkaline Phosphatase Assay Kit (Beyotime Biotechnology, China) according to the manufacturers’ instructions. Para‐nitrophenol (p‐nitrophenol), as a common substrate for phosphatase activity, can produce yellow products under alkaline conditions. Optical density (OD) values were measured using a microplate reader (SpectraMax Plus384, Molecular Devices, USA) at 405 nm. The expression level of ALP was standardized to the total protein content of cells to obtain the absorbance index.

### 2.8 RNA isolation and Quantitative real‐time PCR (qPCR)

In accordance with the manufacturer's instructions, the exosomal RNA was isolated using SeraMir Exosome RNA Purification kit (System Biosciences, USA). TRIzol^™^ Reagent (Invitrogen, USA) and PrimeScript^™^ RT Master Mix (TAKARA, Japan) were used to extract total RNA and synthesize cDNA from cells, respectively. The cycle was as follows: pre‐denaturation at 95°C for 30 seconds; denaturation at 95°C for 5 seconds; and extension at 60°C for 30 seconds. The cycle was done for 40 rounds. All primers were synthesized by GenePharma (China). The primer sequences are given in Table 1. Moreover, the details of primers are provided in Supplementary file 1. GAPDH was used for mRNA normalization. U6 was used for miRNA normalization. The relative expression levels of target genes were calculated using the 2^−ΔΔCt^ method.

**Table 1:** List of gene primers

Gene	Forward sequence	Reverse sequence
Osterix	TTCTGCGGCAAGAGGTTCACTC	GTGTTTGCTCAGGTGGTCGCTT
ALP	CAACGAGGTCATCTCCGTGATG	TACCAGTTGCGGTTCACCGTGT
RUNX2	CCCAGTATGAGAGTAGGTGTCC	GGGTAAGACTGGTCATAGGACC
SIRT7	CAGGGAGTACGTGCGGGTGT	TCGGTCGCCGCTTCCCAGTT
miR‐130a‐3p	CGATGCTCTCAGTGCAATGTTA	
GAPDH	GTCTCCTCTGACTTCAACAGCG	ACCACCCTGTTGCTGTAGCCAA
U6	CGCTTCGGCAGCACATATAC	TTCACGAATTTGCGTGTCATC

### 2.9 Western blot analysis

Cells were lysed by RIPA Lysis Buffer (Beyotime Biotechnology, China). The proteins are electrophoretic separated with 11% SDS‐PAGE gel, transferred to PVDF membrane (Millipore, USA) and stained with Ponceau S staining solution (Beyotime Biotechnology, China) for 5‐10 minutes. After blocking with 5% evaporated skimmed milk, the membranes were incubated with each primary antibody, including anti‐TSG101, anti‐Calnexin, anti‐RUNX2, anti‐Osterix, anti‐Wnt1, anti‐β‐catenin, anti‐Axin2 (Abcam, 1:1000 dilution) for 16 hours and the respective secondary antibody (Cell Signaling Technology, 1:5000 dilution). After the membranes were washed with TBST three times, the target bands were detected by ECL kit (Solarbio, China). The relative intensity was measured by normalization using GAPDH.

### 2.10 Target gene prediction and Dual‐luciferase reporter gene assay

TargetScan (http://www.targetscan.org) online tool was utilized to predict which gave a prediction that miR‐130a‐3p can bind to the 3’UTR of SIRT7. SIRT7 wild‐type vectors, SIRT7 mutant‐type vectors, miR‐130a‐3p vectors and blank vectors were purchased from Genepharma (China). For dual‐luciferase reporter gene assay, HEK293T cells were transiently transfected with SIRT7 wild‐type or SIRT7 mutant‐type plasmids, and miR‐130a‐3p plasmids using Lipofectamine 2000 (Invitrogen). Report gene assay was performed 24 hours after transfection using Dual‐Luciferase Reporter Assay System (Promega, USA). Firefly Luciferase and Ranilla Luciferase were detected by multi‐mode microplate reader (PerkinElmer, USA). Firefly/Ranilla Luciferase activity was used for internal control, and all assays were repeated three times.

### 2.11 Statistical analysis

In this study, SPSS 17.0 and GraphPad Prism 7.0 were used for statistical analysis. Besides, all experiments were repeated at least three times. *P* value < .05 is considered as statistically significant differences.

## 3 RESULTS

### 3.1 Identification of ADSCs

**Figure 1:**
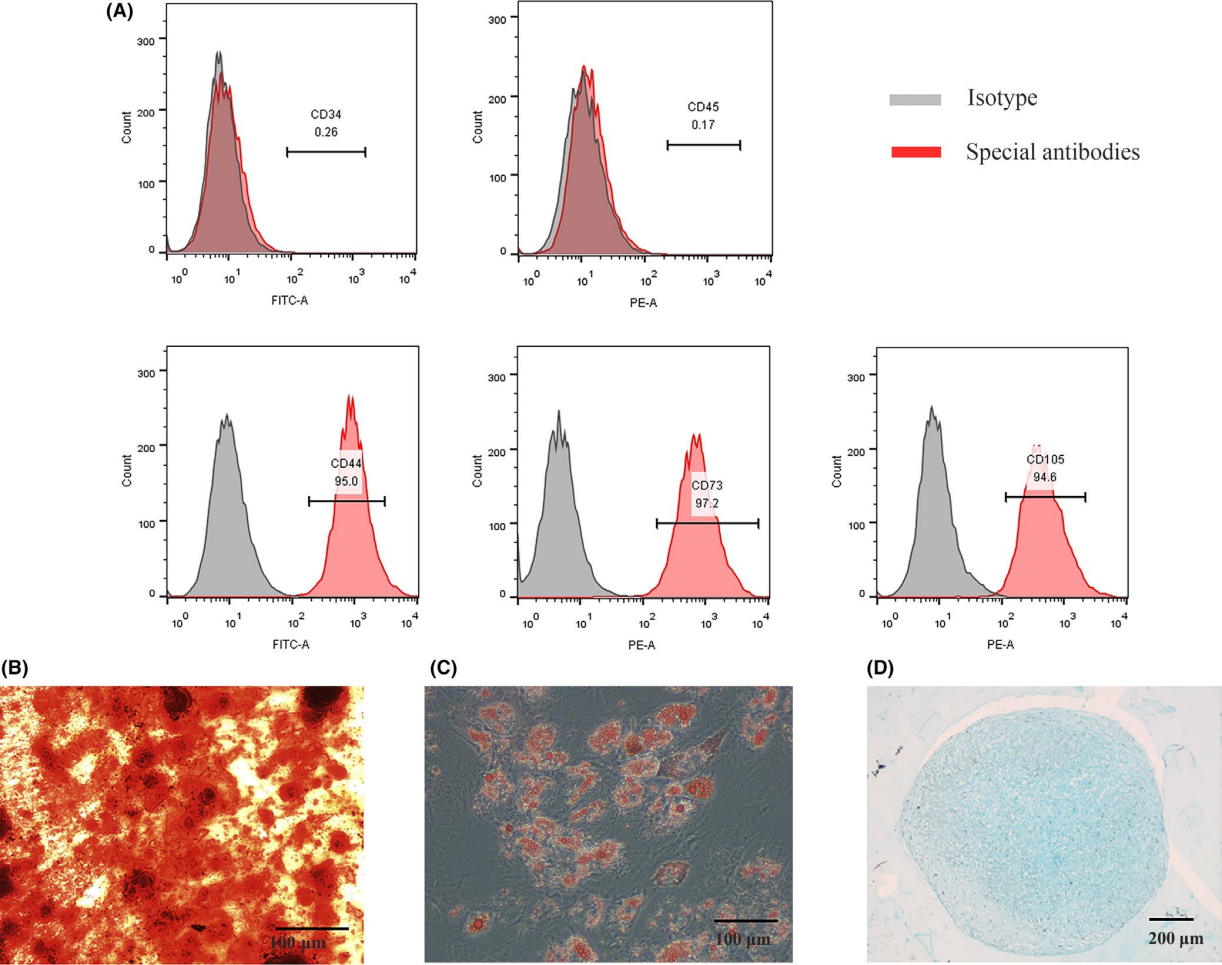
Characteristics of ADSCs. A, The expression of special surface markers detected by flow cytometry analysis. B, The ability of ADSCs to differentiate into osteoblasts confirmed by ARS. C, The ability of ADSCs to differentiate into adipocytes identified by oil red O staining. D, The ability of ADSCs to differentiate into chondrocytes identified by alcian blue. Notes: ARS:Alizarin Red S

To identify characteristics of ADSCs, flow cytometry analysis and multi‐lineage potential assay were performed. As shown in Figure 1(A), the expression of CD44, CD73 and CD105 was positive, while the expression of CD34 and CD45 was negative. In addition, we confirmed that ADSCs can differentiate into adipocytes, osteoblasts and chondrocytes [Figure 1(B‐D)].

### 3.2 Expression of miR‐130a‐3p in ADSCs‐derived exosomes

**Figure 2:**
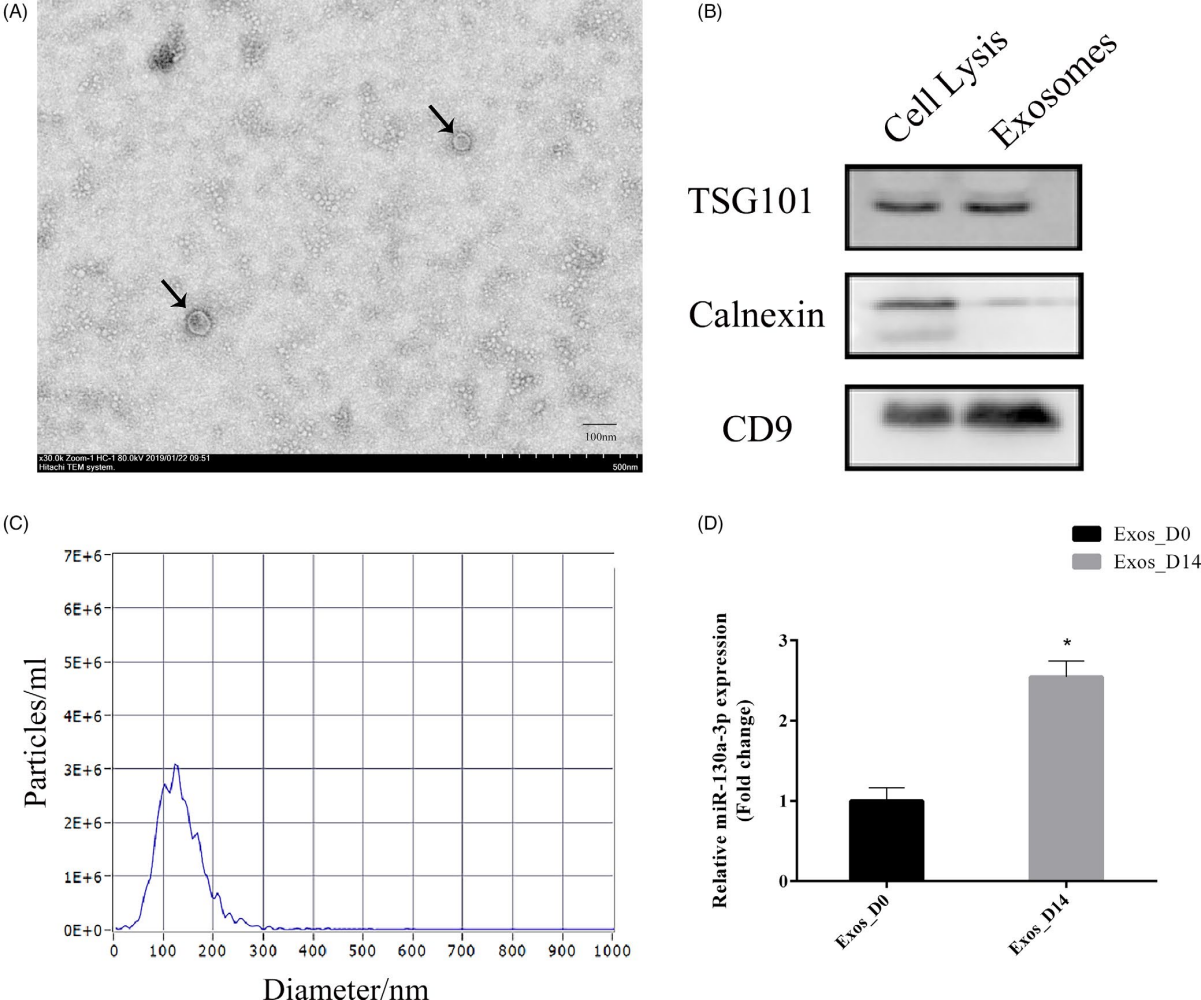
Identification of exosomes derived from ADSCs and the expression of miR‐130a‐3p in ADSCs‐derived exosomes. A, The size and morphology of exosomes observed using TEM. Scale bar: 100 nm. B, Specific markers of exosomes detected by Western blot. C, Nanoparticle tracking analysis of exosomes. D, qPCR analyses expression of miR‐130a‐3p in Exos_D0 and Exos_D14. *represents significant differences between Exos_D0 and Exos_D14. *P < .05

Exosomes derived from undifferentiated ADSCs (Exos_D0) and osteogenically differentiated ADSCs on 14th day (Exos_D14) were extracted by differential ultracentrifugation. As shown in Figure 2(A), the vesicles with a particle size between 40 nm and 150 nm exhibited spherical morphology under TEM, indicating the existence of exosomes. Western blot results showed that the TSG101 and CD9 were positive while calnexin was almost not expressed [Figure 2(B)]. The results of nanoparticle tracking analysis (NTA) revealed that the peak size of exosomes was 119.0 nm, and the peak area per cent was 97.9%. The actual average particles size was 134.2 nm, which conforms to the identification standard of exosomes with particles size of 40‐150 nm [Figure 2(C)]. qPCR results indicated that, compared with Exos‐D0, the expression of miR‐130a‐3p in Exos_D14 was significantly increased[Figure 2(D)].

### 3.3 Overexpression and knockdown of miR‐130a‐3p in ADSCs

**Figure 3:**
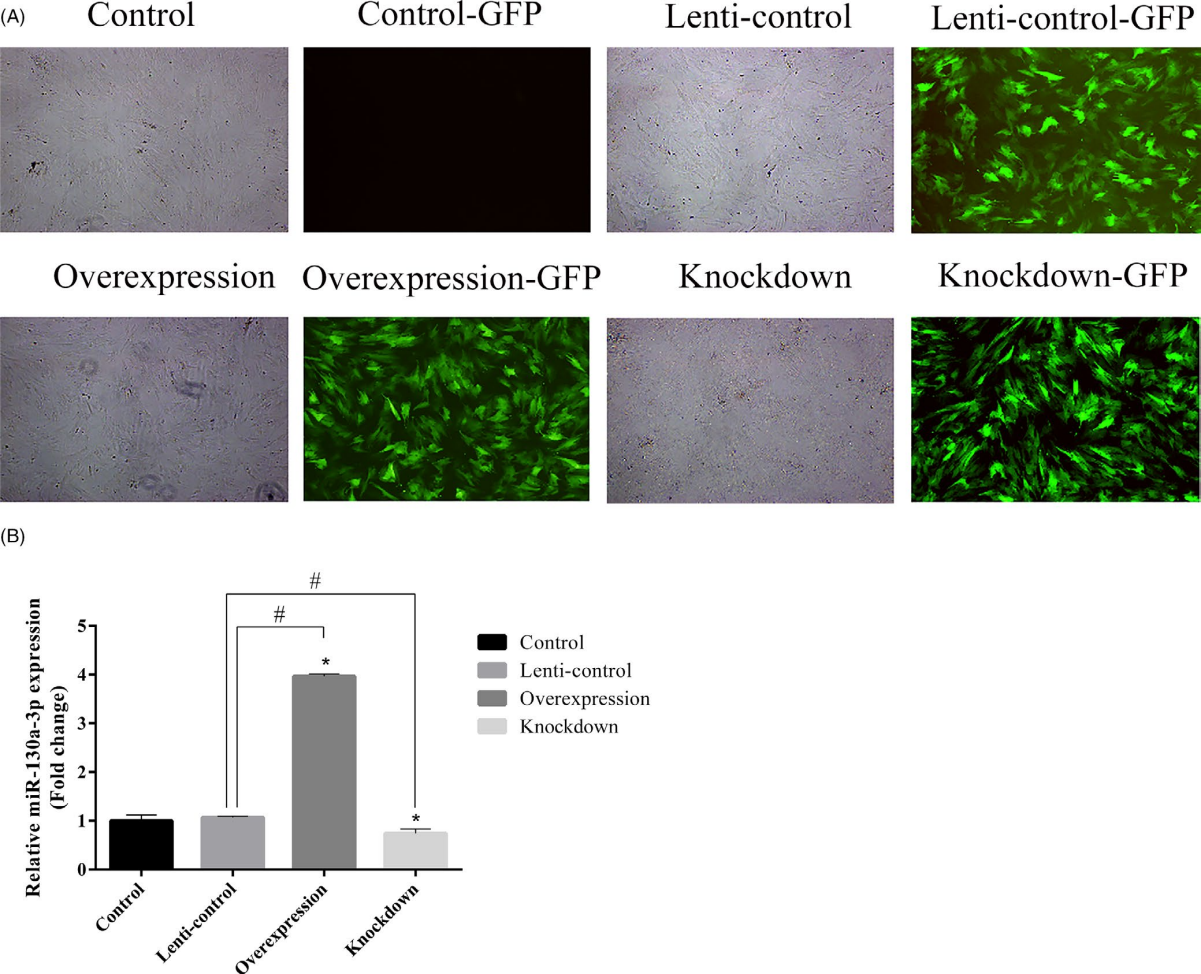
Construction of miR‐130a‐3p overexpressing ADSCs, miR‐130a‐3p knockdown ADSCs and lenti‐control ADSCs. A, 24 h after transfection, ADSCs observed under a normal microscope and an inverted fluorescence microscope (magnification, ×4). B, The expression of miR‐130a‐3p quantified by qPCR. Notes: *represents significant differences between the control group and other groups; #represents significant differences between lenti‐control and overexpression groups, or lenti‐control and knockdown groups. *P < .05; #*P* < .05

To further explore the roles of miR‐130a‐3p, which was highly expressed in Exos_D14, we transfected ADSCs with lentiviral overexpression particles (overexpression group), lentiviral knockdown particles (knockdown group) and lentiviral GFP particles (lenti‐control group), respectively. Most ADSCs in overexpression and knockdown group expressed green fluorescent protein (GFP) whose morphology were similar to that of ADSCs in control group (without lentivirus) and lenti‐control group [Figure 3(A)]. The expression of miR‐130a‐3p was quantified by qPCR. As shown in Figure 3(B), compared with control and lenti‐control groups, miR‐130a‐3p was significantly increased in overexpression group while decreased in knockdown group.

### 3.4 Overexpression of miR‐130a‐3p inhibits proliferation but promotes osteogenic differentiation of ADSCs, while miR‐130a‐3p knockdown promotes proliferation

**Figure 4:**
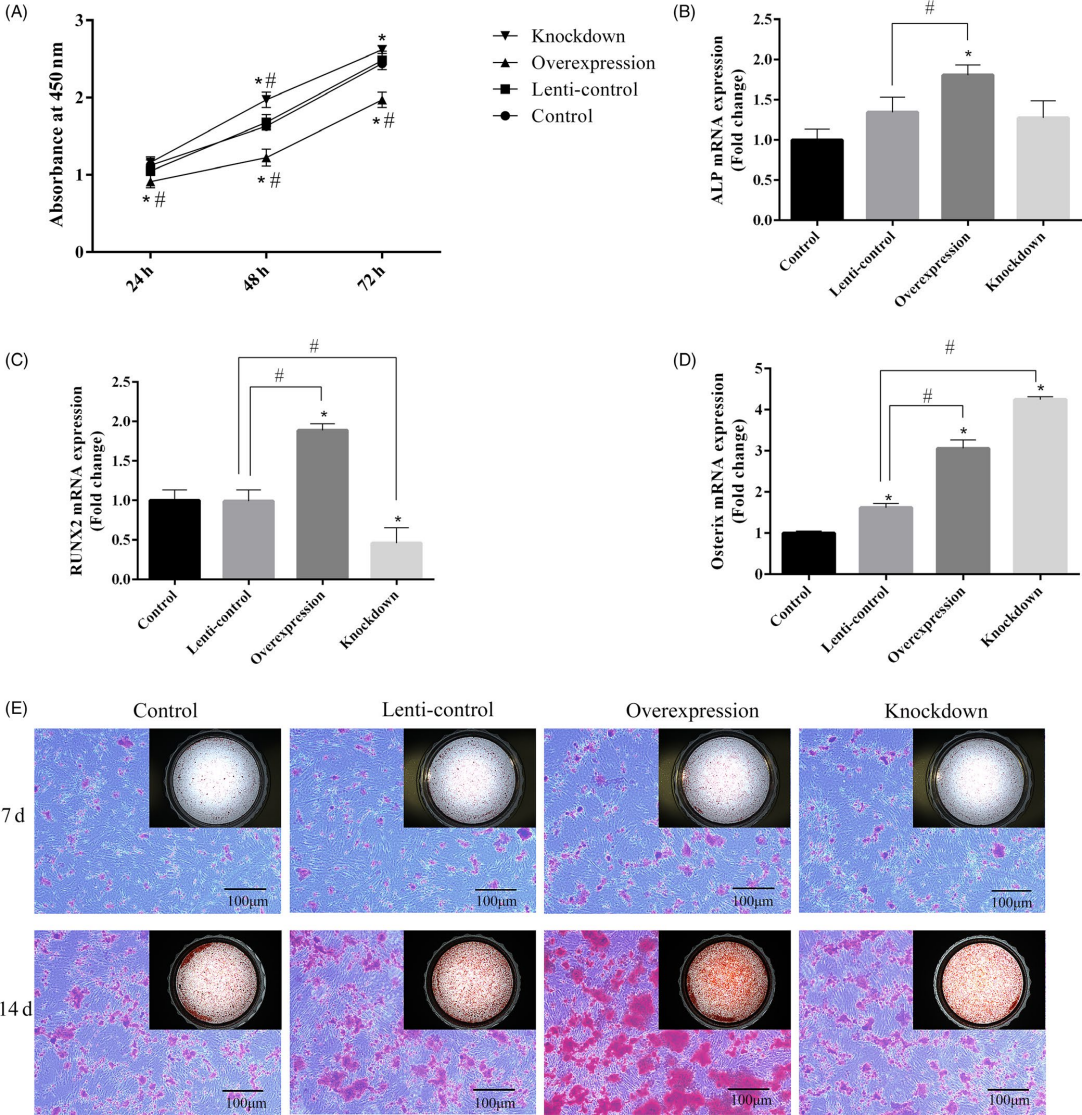
The effect of miR‐130a‐3p on proliferation and differentiation of ADSCs. A, The proliferation rate detected by CCK8. B‐D, The expression of ALP, RUNX2 and Osterix mRNA analysed by qPCR on the third day of induction. E, Calcium deposits detected by ARS assay. Scale bar: 100 μm. Notes: *represents significant differences between control and other groups; #represents significant differences between lenti‐control and overexpression groups, or lenti‐control and knockdown groups. *P < .05; #*P* < .05

To investigate whether miR‐130a‐3p can affect the proliferation of ADSCs, we carried out cell viability assay using CCK8. As the result shown in Figure 4(A), compared with control and lenti‐control group, knockdown of miR‐130a‐3p enhanced proliferation of ADSCs, while overexpression of miR‐130a‐3p inhibited proliferation. There was no significant difference between control and lenti‐control groups.

Furthermore, we performed osteogenic induction of cells in each group to evaluate the roles of miR‐130a‐3p in the osteogenic differentiation of ADSCs. qPCR was performed 3 days after induction [Figure 4(B‐D)]. The expression of ALP, RUNX2 and Osterix mRNA all showed a significant increase in overexpression group compared to control and lenti‐control groups, while the expression of RUNX2 mRNA in knockdown group was dramatically decreased. Surprisingly, the Osterix mRNA expression level in knockdown group was much higher than that in the other groups. After a 7‐day and 14‐day induction, ARS assay indicated that there were more calcium deposits formed in overexpression group [Figure 4(E)]. To sum up, the results confirmed that miR‐130a‐3p can inhibit proliferation while promoting osteogenic differentiation of ADSCs. MiR‐130a‐3p knockdown can promote the proliferation of ADSCs, but the effect of inhibiting osteogenic differentiation is not significant.

### 3.5 MiR‐130a‐3p can target SIRT7 mRNA in the 3’UTR

**Figure 5:**
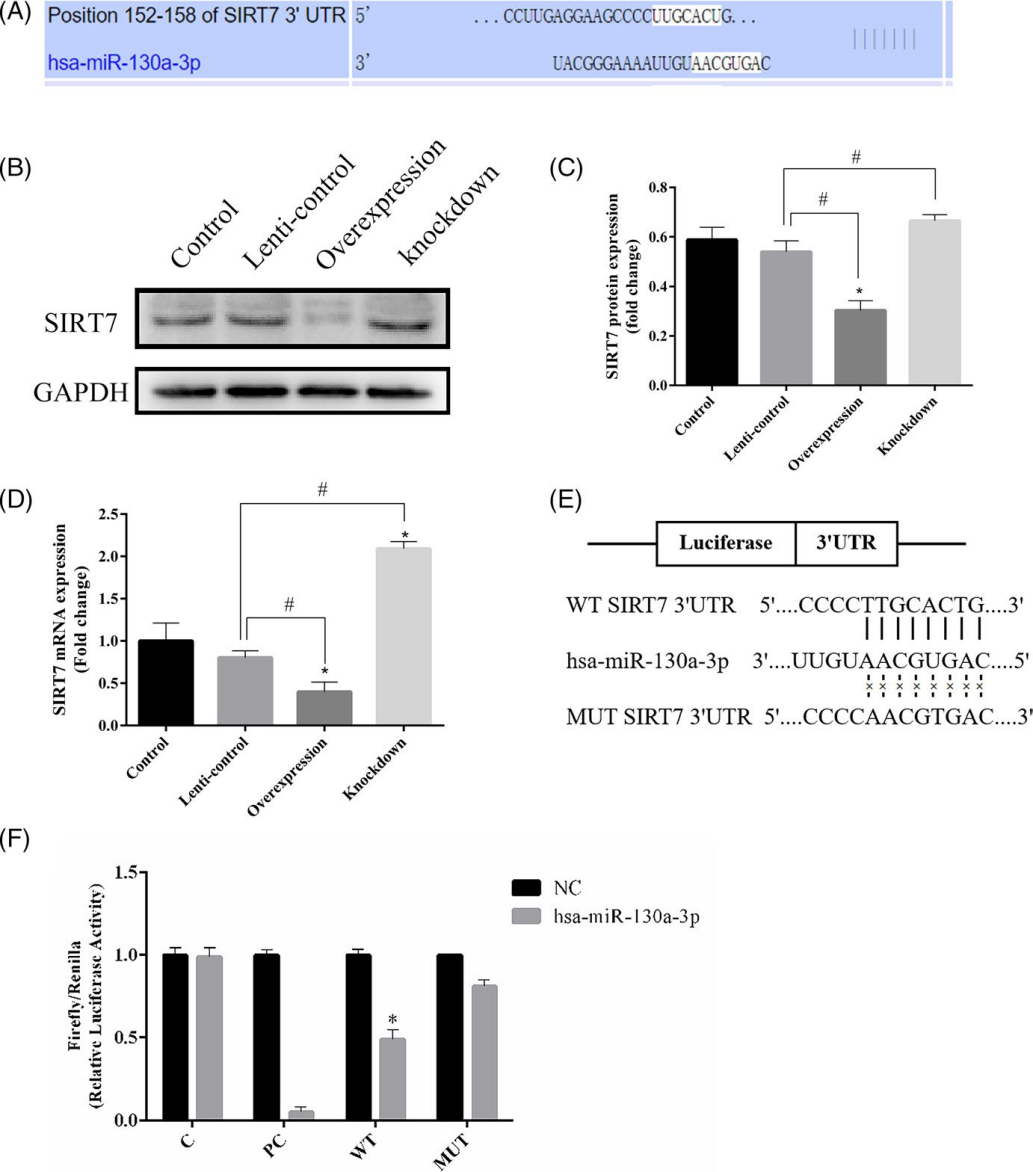
Target binding between miR‐130a‐3p and SIRT7. A, MiR‐130a‐3p predicted to bind to 3’UTR of SIRT7 by bioinformatics analysis. B, The expression of SIRT7 protein analysed by Western blot. C, Relative intensity analyses of Western blot results of SIRT7. D, qPCR performed to detect SIRT7 mRNA. E, 3’UTR of wild‐type SIRT7 mRNA and 3ʹUTR of mutant‐type SIRT7 mRNA cloned into luciferase vectors. F, Luciferase activity was detected. Notes: C: blank control; PC: miR‐130a‐3p positive control; WT:SIRT7 wild type; MUT:SIRT7 mutant type; NC: negative control. *represents significant differences between control and other groups; #represents significant differences between lenti‐control and overexpression groups, or lenti‐control and knockdown groups; and *represents significant differences between NC and hsa‐miR‐130a‐3p groups. *P < .05, #*P* < .05, *P < .05

In order to explore how miR‐130a‐3p regulates osteogenic differentiation of ADSCs, we searched for its potential downstream target genes through bioinformatics analysis. TargetScan tool predicted that miR‐130a‐3p can bind to the 3’UTR of SIRT7 [Figure 5(A)]. As shown in Figure 5(B,C), overexpression of miR‐130a‐3p in ADSCs resulted in the dramatically decrease of SIRT7 protein expression, while knockdown of miR‐130a‐3p in ADSCs resulted in the significantly increase of SIRT7 protein expression according to Western blot analysis. qPCR analysis showed similar results to Western blot analysis [Figure 5(D)]. To further determine the negative regulatory effect of miR‐130a‐3p on SIRT7, 3’UTR of wild‐type SIRT7 mRNA (WT) and 3ʹUTR of mutant‐type SIRT7 mRNA (MUT) were cloned into luciferase vectors for dual‐luciferase reporter gene assay [Figure 5(E)]. According to the result of dual‐luciferase reporter, we discovered that luciferase activity was significantly decreased due to overexpression of miR‐130a‐3p in HEK293T cells with SIRT7 wild‐type vectors [Figure 5(F)]. In summary, our results confirmed that miR‐130a‐3p negatively regulates expression of SIRT7 by targeting its 3’UTR.

### 3.6 Exosomal miR‐130a‐3p regulates osteogenic differentiation of ADSCs by mediating Wnt signalling pathway

**Figure 6:**
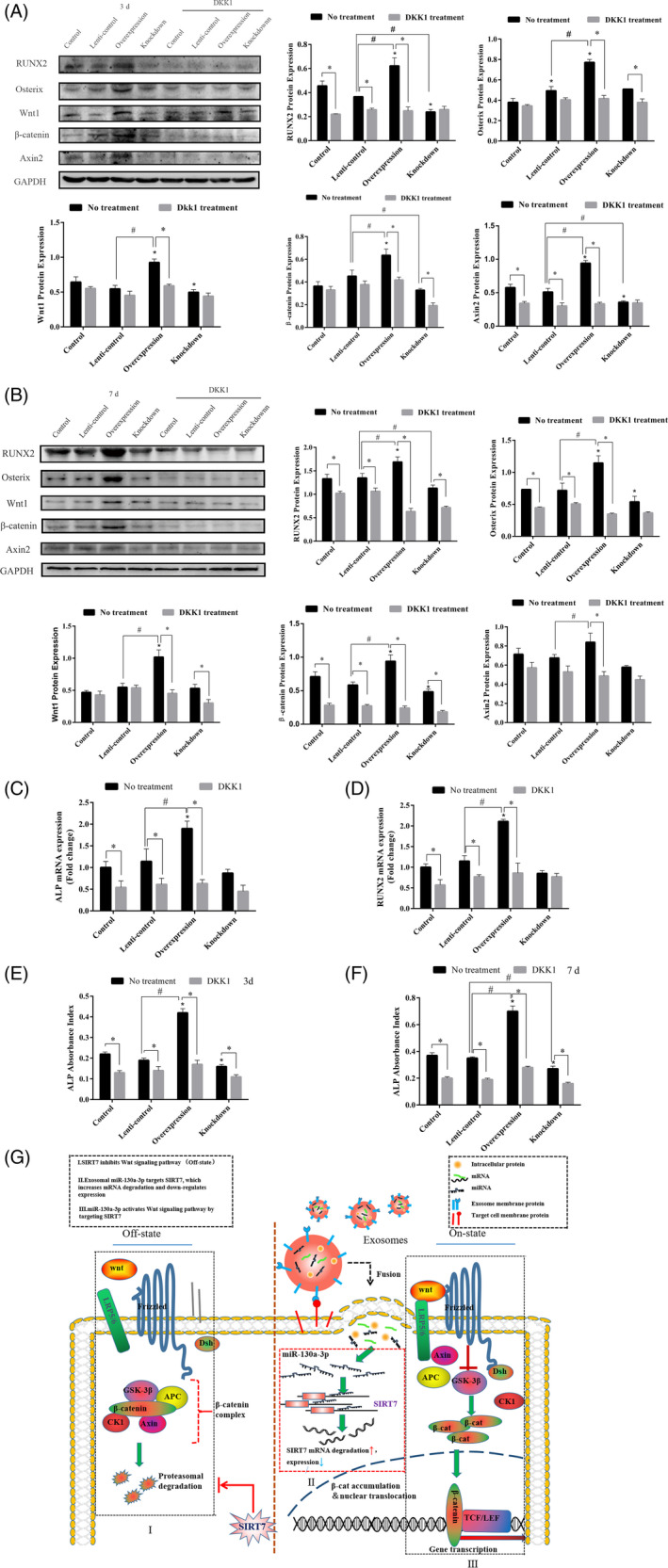
The mechanism of miR‐130a‐3p promoting osteogenic differentiation of ADSCs through Wnt signalling pathway. A‐B, The expression of osteogenic‐related protein and key protein of Wnt signalling pathway analysed by Western blot on 3rd and 7th day. C‐D, The expression of ALP and RUNX2 mRNA detected by qPCR. E‐F, ALP activity detected by ALP assay kit. G, The diagram of the mechanism that exosomal miR‐130a‐3p regulates osteogenic differentiation of ADSCs by mediating Wnt signalling pathway. Notes:*represents significant differences between control and other groups without DKK1 treatment; *represents significant differences between no treatment and DKK1 treatment group; and #represents significant differences between lenti‐control and overexpression groups, or lenti‐control and knockdown groups without DKK1 treatment. *P < .05; *P < .05; #*P* < .05

To further clarify the specific mechanism by which miR‐130a‐3p regulated osteogenic differentiation of ADSCs, the Wnt signalling pathway knockdown groups were added based on the original experimental grouping, namely control + DKK1, lenti‐control + DKK1, overexpression + DKK1 and knockdown + DKK1. Western blots were performed on Day 3 and Day 7 after treatment [Figure 6(A‐B)]. In the comparison of first four groups, we found that the expression of osteogenic‐related proteins RUNX2, Osterix and key proteins of Wnt signalling pathway Wnt1, β‐catenin and Axin2 in the overexpression group were significantly increased. Compared with the first four groups, the osteogenic‐related proteins and proteins of Wnt signalling pathway in the last four groups have a downward trend. In the comparison between overexpression group and overexpression + DKK1 group, we found that the expression of osteogenic‐related protein (RUNX2, Osterix) of the latter decreased more significantly.

As shown by the qPCR results after a 7‐day treatment, we found that compared with control and lenti‐control groups, the expression of ALP and RUNX2 mRNA in overexpression group increased significantly without DKK1. While there were no significant differences in knockdown group. Compared with no treatment groups, the expression of ALP and RUNX2 mRNA in control, lenti‐control and overexpression groups treated with DKK1 decreased significantly. Additionally, the differences of ALP and RUNX2 mRNA expression in knockdown group were not significant. Among them, the expression of ALP and RUNX2 in the overexpression group was most significantly decreased after DKK1 treatment [Figure 6(C,D)].

As the result shown in Figure 6(E,F), compared with control and lenti‐control groups, ALP activity on 3rd day and 7th day was significantly increased in overexpression group without DKK1. Compare with control group, ALP activity on 3rd day was significantly decreased. ALP activity on 7th day was significantly decreased, compared with control and lenti‐control groups. Compared with no DKK1 treatment groups, ALP activity in groups with DKK1 treatment was significantly decreased. ALP activity in the overexpression group was most significantly decreased. The diagram of the mechanism that exosomal miR‐130a‐3p regulates osteogenic differentiation of ADSCs by mediating Wnt signalling pathway was presented in Figure 6(G).

In general, these results confirmed that blocking Wnt signalling pathway inhibits osteogenesis and further proved that miR‐130a‐3p promotes osteogenic differentiation of ADSCs by activating Wnt signalling pathway.

## 4 DISCUSSION

It is an urgent issue in the field of bone regeneration to find an efficient way to induce osteogenic differentiation of stem cells. Our previous study has confirmed that only osteogenically differentiated ADSC‐derived exosomes can promote osteogenic differentiation of ADSCs, which is expected to be an effective osteogenic induction method.^4^ To explore how exosomes worked, we selected miR‐130a‐3p, which is highly expressed in exosomes derived from osteogenically differentiated ADSC, for subsequent study. Seenprachwwong et al have proven that miR‐130a and miR‐27b can enhance hBMSCs via specific down‐regulation of PPAR‐γ (a major transcription factor of adipogenesis).^28^ It suggested that the osteogenic differentiation of MSCs is negatively correlated with adipogenesis. Hence, osteogenic potential of MSCs could be enhanced by inhibiting their adipogenesis. In the current study, we clarified the underlying mechanism of miR‐130a‐3p in regulating proliferation and osteogenic differentiation of ADSCs by altering its expression. Importantly, exosomes may modulate proliferation and osteogenic differentiation of ADSCs by delivering miR‐130a‐3p.

The fact that exosomes carry miRNAs which serve as important mediators of intercellular communication, has been proven to promote osteogenic differentiation of stem cells.^4^, ^29^, ^30^ MiRNAs play a crucial role in bone development and homoeostasis.^8^, ^31^ Our findings revealed that overexpression of miR‐130a‐3p can promoted osteogenic differentiation but inhibited proliferation of ADSCs. Knockdown of miR‐130a‐3p can promoted proliferation without significantly inhibiting osteogenic differentiation of ADSCs. Consistently, it has been reported that miR‐130a‐3p inhibited cell proliferation in nasopharyngeal carcinoma.^32^ Additionally, lipid production was decreased due to 3T3‐L1 cells being treated with miR‐130a‐3p.^10^ To our knowledge, this is the first demonstration of the important role of exosomal miR‐130a‐3p in regulating osteogenic differentiation and proliferation of ADSCs.

Furthermore, our results also indicated that miR‐130a‐3p can target SIRT7 by dual‐luciferase reporter gene assay. Knockdown of SIRT7 using lentiviral transfection is now confirmed to promote osteogenic differentiation of bone marrow mesenchymal stem cells.^20^ However, there are safety risks in the clinical application of lentiviral modified cells. In this study, we explored the possibility of miR‐130a‐3p enriched in safer exosomes as SIRT7 targeting suppressor, to achieve the similar effect of promoting osteogenic differentiation of ADSCs, which is more significant for clinical application of promoting bone regeneration.

In this study, we found that miR‐130a‐3p overexpression can dramatically increase the expression of ALP, RUNX2 and Osterix mRNA, Osterix and RUNX2 protein. Interestingly, although miR‐130a‐3p overexpression increased the expression of Osterix mRNA, in comparison, miR‐130a‐3p knockdown can more significantly increase it. According to previous study, SIRT7 increased the transcriptional activity of Osterix without altering protein expression,^33^ which further confirmed the role of SIRT7 in miR‐130a‐3p regulating osteogenic differentiation. Moreover, there may be other pathways affecting the expression of Osterix mRNA and protein which may explain why its expression increases with the decrease of SIRT7 (miR‐130a‐3p overexpression group) during osteogenic differentiation. ARS clearly showed that miR‐130a‐3p overexpression increased calcium deposits. These results indicated that miR‐130a‐3p overexpression can promote osteogenic differentiation of ADSCs.

The canonical Wnt signalling pathway plays an important role in osteogenic differentiation of stem cells.^34^ It is reported that several members of the sirtuin family are closely related to Wnt signalling pathway.^20^, ^35^, ^36^ In this study, we found that overexpression of miR‐130a‐3p can decrease SIRT7 expression while increasing β‐catenin and Axin2 expression during osteogenic differentiation. After adding DKK1 (wnt signalling pathway inhibitor), we found that the expression of osteogenesis‐related genes and proteins in the miR‐130a‐3p overexpression group was most significant in decrease, whereas being not significant in decrease in miR‐130a‐3p knockdown group. All in all, the effect of miR‐130a‐3p overexpression on osteogenic differentiation of ADSCs was partially reversed by DKK1 (an inhibitor of Wnt signalling pathway). These results suggested that miR‐130a‐3p overexpression positively regulated osteogenic differentiation of ADSCs partly through modulating Wnt/β‐catenin signalling pathway.

More and more studies have confirmed that exosome plays an important role in osteogenesis.^37^, ^38^ In our present study, for the first time, we confirmed the effect of exosomal miR‐130a‐3p on proliferation and osteogenic differentiation of ADSCs. These findings indicated that exosomal miR‐130a‐3p may be a new target for bone regeneration. Other potential targets of miR‐130a‐3p and signalling pathways involved in regulating osteogenic differentiation by exosomal miR‐130a‐3p should be explored in the future. Additionally, the function of more miRNAs delivered through exosomes which is related to osteogenesis remains to be elucidated.

Long‐term vision, the real achieve clinical application of the method by exosomal miRNAs to treat bone defects, there is still a long way to go. The development of bone tissue engineering provides a shortcut. Bone tissue engineering is mainly composed of three elements: seed cells, inducible factors and scaffold materials. In research experiments, we confirmed that exosomal miR‐130a‐3p can be used as a highly effective osteogenic induction factor in the construction of tissue engineering bone. At the same time, we need a scaffold material can provide 'matrix' support to solve the problem that it cannot act alone on local bone defects. Therefore, the preparation of scaffold materials with good structure, strength and other properties that can be used in vivo is an urgent problem to be solved at present. It will be the focus of future research, which will also greatly promote the development of clinical treatment of bone defects.

## 5 CONCLUSIONS

In conclusion, our results showed that exosomal miR‐130a‐3p can regulate proliferation and osteogenic differentiation of ADSCs. The overexpression of miR‐130a‐3p promoted osteogenic differentiation but inhibited proliferation, while knockdown only promoted proliferation of ADSCs. Thus, exosomes rich in miR‐130a‐3p may become a novel 'inducible factor' to promote regeneration and repair in the future.

## CONFLICT OF INTEREST

No conflicts of interest exist.

## AUTHORS’ CONTRIBUTIONS

Shude Yang carried out all the experiments, collection and analysis of data, and wrote the manuscript. Shu Guo performed experimental guidance and data analysis. Shuang Tong and Xu Sun contributed to collection of human adipose tissue. All authors read and approved the final manuscript.

## ETHICAL APPROVAL

The study is approved by the Ethics Committee of the First Hospital of China Medical University, Shenyang, China (No.2018‐110‐2).

## Supporting information

Supplementary Material

## Data Availability

All data obtained or analysed in this study are included in this paper.

## References

[1] Pittenger MF , Mackay AM , Beck SC , et al. Multilineage potential of adult human mesenchymal stem cells. Science. 1999;284(5411):143‐147. 10102814 10.1126/science.284.5411.143

[2] Tapp H , Hanley EN , Patt JC , Gruber HE . Adipose‐derived stem cells: characterization and current application in orthopaedic tissue repair. Exp Biol Med (Maywood). 2009;234(1):1‐9. 19109553 10.3181/0805/MR-170

[3] Rada T , Reis RL , Gomes ME . Adipose tissue‐derived stem cells and their application in bone and cartilage tissue engineering. Tissue Eng Part B Rev. 2009;15(2):113‐125. 19196117 10.1089/ten.teb.2008.0423

[4] Yang SD , Guo S , Tong S , Sun X . Promoting osteogenic differentiation of human adipose‐derived stem cells by altering the expression of exosomal miRNA. Stem Cells Int. 2019;1351860. 31354836 10.1155/2019/1351860 PMC6636464

[5] Bartel DP . MicroRNAs: genomics, biogenesis, mechanism, and function. Cell. 2004;116(2):281‐297. 14744438 10.1016/s0092-8674(04)00045-5

[6] Valadi H , Ekström K , Bossios A , Sjöstrand M , Lee JJ , Lötvall JO . Exosome‐mediated transfer of mRNAs and microRNAs is a novel mechanism of genetic exchange between cells. Nat Cell Biol. 2007;9(6):654‐659. 17486113 10.1038/ncb1596

[7] Chen S , Tang Y , Liu Y , et al. Exosomes derived from miR‐375‐overexpressing human adipose mesenchymal stem cells promote bone regeneration. Cell Prolif. 2019;52(5):e12669. 31380594 10.1111/cpr.12669 PMC6797519

[8] Dole NS , Delany AM . MicroRNA variants as genetic determinants of bone mass. Bone. 2016;84:57‐68. 26723575 10.1016/j.bone.2015.12.016 PMC4755870

[9] Xiao F , Yu J , Liu B , et al. A novel function of microRNA 130a–3p in hepatic insulin sensitivity and liver steatosis. Diabetes. 2014;63(8):2631‐2642. 24677715 10.2337/db13-1689

[10] Wu J , Dong T , Chen T , et al. Hepatic exosome‐derived miR‐130a‐3p attenuates glucose tolerance via suppressing PHLPP2 gene in adipocyte. Metabolism. 2019;103:154006. 31715176 10.1016/j.metabol.2019.154006

[11] Hu B , Zhang H , Wang Z , Zhang F , Wei H , Li L . LncRNA CCAT1/miR‐130a‐3p axis increases cisplatin resistance in non‐small‐cell lung cancer cell line by targeting SOX4. Cancer Biol Ther. 2017;18(12):974‐983. 29020498 10.1080/15384047.2017.1385679 PMC5718806

[12] Yu XF , Wang J , Ouyang N , et al. The role of miR‐130a‐3p and SPOCK1 in tobacco exposed bronchial epithelial BEAS‐2B transformed cells: comparison to A549 and H1299 lung cancer cell lines. J Toxicol Environ Health A. 2019;82(15):862‐869. 31526129 10.1080/15287394.2019.1664479

[13] Blank MF , Grummt I . The seven faces of SIRT7. Transcription. 2017;8(2):67‐74. 28067587 10.1080/21541264.2016.1276658 PMC5423475

[14] Yu J , Qin B , Wu F , et al. Regulation of serine‐threonine kinase Akt activation by NAD+‐dependent deacetylase SIRT7. Cell Rep. 2017;18(5):1229‐1240. 28147277 10.1016/j.celrep.2017.01.009 PMC5298804

[15] Houtkooper RH , Pirinen E , Auwerx J . Sirtuins as regulators of metabolism and healthspan. Nat Rev Mol Cell Biol. 2012;13(4):225‐238. 22395773 10.1038/nrm3293 PMC4872805

[16] Cohen‐Kfir E , Artsi H , Levin A , et al. Sirt1 is a regulator of bone mass and a repressor of Sost encoding for sclerostin, a bone formation inhibitor. Endocrinology. 2011;152(12):4514‐4524. 21952235 10.1210/en.2011-1128

[17] Ding Y , Yang H , Wang Y , Chen J , Ji Z , Sun H . Sirtuin 3 is required for osteogenic differentiation through maintenance of PGC‐1α‐SOD2‐mediated regulation of mitochondrial function. Int J Biol Sci. 2017;13(2):254‐264. 28255277 10.7150/ijbs.17053 PMC5332879

[18] Sun H , Wu Y , Fu D , Liu Y , Huang C . SIRT6 regulates osteogenic differentiation of rat bone marrow mesenchymal stem cells partially via suppressing the nuclear factor‐kappaB signaling pathway. Stem Cells. 2014;32(7):1943‐1955. 24510807 10.1002/stem.1671

[19] Fang J , Ianni A , Smolka C , et al. Sirt7 promotes adipogenesis in the mouse by inhibiting autocatalytic activation of Sirt1. Proc Natl Acad Sci U S A. 2017;114(40):E8352‐E8361. 28923965 10.1073/pnas.1706945114 PMC5635888

[20] Chen EEM , Zhang W , Ye CCY , et al. Knockdown of SIRT7 enhances the osteogenic differentiation of human bone marrow mesenchymal stem cells partly via activation of the Wnt/β‐catenin signaling pathway. Cell Death Dis. 2017;8(9):e3042. 28880264 10.1038/cddis.2017.429 PMC5636975

[21] Kim JK , Noh JH , Jung KH , et al. Sirtuin7 oncogenic potential in human hepatocellular carcinoma and its regulation by the tumor suppressors MiR‐125a‐5p and MiR‐125b. Hepatology. 2013;57(3):1055‐1067. 23079745 10.1002/hep.26101

[22] Nguyen P , Lee S , Lorang‐Leins D , Trepel J , Smart DK . SIRT2 interacts with beta‐catenin to inhibit Wnt signaling output in response to radiation‐induced stress. Mol Cancer Res. 2014;12(9):1244‐1253. 24866770 10.1158/1541-7786.MCR-14-0223-T PMC4163538

[23] Wang H , Diao D , Shi Z , et al. Ju Z (2016) SIRT6 controls hematopoietic stem cell homeostasis through epigenetic regulation of Wnt signaling. Cell Stem Cell. 2016;18(4):495‐507. 27058938 10.1016/j.stem.2016.03.005

[24] Clevers H , Loh KM , Nusse R . Stem cell signaling. An integral program for tissue renewal and regeneration: Wnt signaling and stem cell control. Science. 2014;346(6205):1248012. 25278615 10.1126/science.1248012

[25] Zuk PA , Zhu M , Mizuno H , et al. Multilineage cells from human adipose tissue: implications for cell‐based therapies. Tissue Eng. 2001;7(2):211‐228. 11304456 10.1089/107632701300062859

[26] Bunnell BA , Flaat M , Gagliardi C , Patel B , Ripoll C . Adipose‐derived stem cells: isolation, expansion and differentiation. Methods. 2008;45(2):115‐120. 18593609 10.1016/j.ymeth.2008.03.006 PMC3668445

[27] Théry C , Amigorena S , Raposo G , Clayton A . Isolation and characterization of exosomes from cell culture supernatants and biological fluids. Curr Protoc Cell Biol. 2006; Chapter 3, Unit 3.22. 10.1002/0471143030.cb0322s30 18228490

[28] Seenprachawong K , Tawornsawutruk T , Nantasenamat C , et al. miR‐130a and miR‐27b enhance osteogenesis in human bone marrow mesenchymal stem cells via specific down‐regulation of peroxisome proliferator‐activated receptor γ. Front Genet. 2018;9:1–15. 30487813 10.3389/fgene.2018.00543 PMC6246628

[29] Narayanan R , Huang C‐C , Ravindran S . Hijacking the cellular mail: exosome mediated differentiation of mesenchymal stem cells. Stem Cells Int. 2016;2016:1‐11. 10.1155/2016/3808674 PMC4736778 26880957

[30] Fang S , Li Y , Chen P . Osteogenic effect of bone marrow mesenchymal stem cell‐derived exosomes on steroid‐induced osteonecrosis of the femoral head. Drug Des Devel Ther. 2018;13:45‐55. 10.2147/DDDT.S178698 PMC6305133 30587927

[31] Waki T , Lee SY , Niikura T , et al. Profiling microRNA expression during fracture healing. BMC Musculoskelet Disord. 2016;17:83. 26879131 10.1186/s12891-016-0931-0 PMC4754871

[32] Qu R , Sun Y , Li Y , et al. MicroRNA‐130a‐3p suppresses cell viability, proliferation and invasion in nasopharyngeal carcinoma by inhibiting CXCL12. Am J Transl Res. 2017;9(8):3586‐3598. 28861150 PMC5575173

[33] Fukuda M , Yoshizawa T , Karim MF , et al. SIRT7 has a critical role in bone formation by regulating lysine acylation of SP7/Osterix. Nat Commun. 2018;9(1):2833. 30026585 10.1038/s41467-018-05187-4 PMC6053369

[34] Yuan Z , Li Q , Luo S , et al. PPARγ and Wnt signaling in adipogenic and osteogenic differentiation of mesenchymal stem cells. Curr Stem Cell Res Ther. 2016;11(3):216‐225. 25986621 10.2174/1574888x10666150519093429

[35] Iyer S , Han L , Bartell SM , et al. Sirtuin (Sirt1) promotes cortical bone formation by preventing β‐catenin sequestration by FoxO transcription factors in osteoblast progenitors. J Biol Chem. 2014;289(35):24069‐24078. 25002589 10.1074/jbc.M114.561803 PMC4148840

[36] Wang H , Diao D , Shi Z , et al. SIRT 6 controls hematopoietic stem cell homeostasis through epigenetic regulation of Wnt signaling. Cell Stem Cell. 2016;18(4):495‐507. 27058938 10.1016/j.stem.2016.03.005

[37] Xu JF , Yang GH , Pan XH , et al. Altered microRNA expression profile in exosomes during osteogenic differentiation of human bone marrow‐derived mesenchymal stem cells. PLoS One. 2014;9(12):e114627. 25503309 10.1371/journal.pone.0114627 PMC4263734

[38] Liao W , Ning Y , Xu HJ , et al. BMSC‐derived exosomes carrying microRNA‐122‐5p promote proliferation of osteoblasts in osteonecrosis of the femoral head. Clin Sci. 2019;133(18):1955‐1975. 10.1042/CS20181064 31387936

