# Supplementary material for: Exosomal miR‐130a‐3p regulates osteogenic differentiation of Human Adipose‐Derived stem cells through mediating SIRT7/Wnt/β‐catenin axis
Source: Cell Prolif. 2020 Aug 17;53(10):e12890. doi: 10.1111/cpr.12890 (PMC7574877; doi:10.1111/cpr.12890)
Supplement: Supplementary file 1 — Supplementary Material [file CPR-53-e12890-s001.docx]

Gene: Osterix

Forward sequence: TTCTGCGGCAAGAGGTTCACTC

Tm: 63.1 GC%:54.55

Reverse sequence: GTGTTTGCTCAGGTGGTCGCTT

Tm: 63.8 GC%:54.55

Product length：129

Gene: ALP

Forward sequence: CAACGAGGTCATCTCCGTGATG

Tm: 61.1 GC%:54.55

Reverse sequence: TACCAGTTGCGGTTCACCGTGT

Tm: 64.6 GC%:54.55

Product length：129

Gene: RUNX2

Forward sequence: CCCAGTATGAGAGTAGGTGTCC

Tm: 59.0 GC%:54.55

Reverse sequence: GGGTAAGACTGGTCATAGGACC

Tm: 59.3 GC%:54.55

Product length：149

Gene: SIRT7

Forward sequence: CAGGGAGTACGTGCGGGTGT

Tm: 64.3 GC%:65.00

Reverse sequence: TCGGTCGCCGCTTCCCAGTT

Tm: 66.7 GC%:65.00

Product length：165

Gene: miR-130a-3p

Forward sequence: CGATGCTCTCAGTGCAATGTTA

Tm: 57.7 GC%:45.45

Product length：83

Gene: GAPDH

Forward sequence: GTCTCCTCTGACTTCAACAGCG

Tm: 60.9 GC%:54.55

Reverse sequence: ACCACCCTGTTGCTGTAGCCAA

Tm: 64.4 GC%:54.55

Product length：131

Gene: U6

Forward sequence: CGCTTCGGCAGCACATATAC

Tm:58.8 GC%:55.00

Reverse Sequence: TTCACGAATTTGCGTGTCATC

Tm: 58.2 GC%:42.86

Product length：87
